# Obituary

**Published:** 1874-08

**Authors:** Thomas H. Chandler


### Editorial.

### OBITUARY.

Died on the night of June 24th, of Meningitis complicated with Pneumonia, Professor T. B. Hitchcock, of the Dental School of Harvard University.

This will be sad news to the dental profession throughout this country. Dr. Hitchcock occupied a very responsible position, and was fulfilling its duties most fully, and so well had his professional work been done thus far, that great expectations were entertained in respect to his work in the future. The profession, and Harvard Dental School, have in his death sustained a loss that can hardly be repaired, and his family and society an irretrievable loss. The family will have the deep and heartfelt sympathy of all who knew him.

The Faculty of which he was a member took the following action:

THE LATE DR. HITCHCOCK.

At a meeting of the faculty of the Dental School of Harvard University, President Eliot presiding, the following resolutions were adopted, viz:

*Resolved*, That the faculty of the Dental School of Harvard University have been deeply grieved at the death of their dean, Dr. Thomas Barnes Hitchcock, and in recognition of his character and services, deem it their duty to place on record their regret for his loss and their sense of his merit.

*Resolved*, That in him the Harvard Dental School has lost a valuable officer, whose unwearied and successful discharge of the duties of his professorship and unselfish interest in his work as dean entitle him to the respect and gratitude of all who are interested in the cause of dental education.

*Resolved*, That the dean be directed to communicate a copy of these resolutions to the family of the deceased, with assurances of our sincere sympathy in their bereavement.

THOMAS H. CHANDLER, Dean.

